# A novel large deletion of the ICR1 region including *H19* and putative enhancer elements

**DOI:** 10.1186/s12881-015-0173-2

**Published:** 2015-05-06

**Authors:** Helen Fryssira, Stella Amenta, Deniz Kanber, Christalena Sofocleous, Evangelia Lykopoulou, Christina Kanaka-Gantenbein, Flavia Cerrato, Hermann-Josef Lüdecke, Susanne Bens, Andrea Riccio, Karin Buiting

**Affiliations:** Aghia Sophia Children’s Hospital, University of Athens Medical School, Athens, Goudi 11527 Greece; Mitera Maternity Hospital, Athens, Greece; Institute of Human Genetics, University Hospital Essen, University Duisburg-Essen, Essen, Germany; First Pediatric Clinic, Aghia Sophia” Children’s Hospital, University of Athens School of Medicine, Athens, Greece; Institute of Genetics and Biophysics A. Buzzati-Traverso, CNR, DiSTABiF, 2nd University of Naples, Naples, Italy; Institute of Human Genetics, University Hospital Schleswig-Hostein Campus Kiel/Christian-Albrechts University Kiel, Kiel, Germany

**Keywords:** Beckwith-Wiedemann syndrome, Genomic imprinting, Imprinting disorders, DNA methylation

## Abstract

**Background:**

Beckwith-Wiedemann syndrome (BWS) is a rare pediatric overgrowth disorder with a variable clinical phenotype caused by deregulation affecting imprinted genes in the chromosomal region 11p15. Alterations of the imprinting control region 1 (ICR1) at the *IGF2/H19* locus resulting in biallelic expression of *IGF2* and biallelic silencing of *H19* account for approximately 10% of patients with BWS. The majority of these patients have epimutations of the ICR1 without detectable DNA sequence changes. Only a few patients were found to have deletions. Most of these deletions are small affecting different parts of the ICR1 differentially methylated region (ICR1-DMR) removing target sequences for CTCF. Only a very few deletions reported so far include the *H19* gene in addition to the CTCF binding sites. None of these deletions include *IGF2*.

**Case presentation:**

A male patient was born with hypotonia, facial dysmorphisms and hypoglycemia suggestive of Beckwith-Wiedemann syndrome. Using methylation-specific (MS)-MLPA (Multiplex ligation-dependent probe amplification) we have identified a maternally inherited large deletion of the ICR1 region in a patient and his mother. The deletion results in a variable clinical expression with a classical BWS in the mother and a more severe presentation of BWS in her son. By genome-wide SNP array analysis the deletion was found to span ~100 kb genomic DNA including the ICR1DMR, *H19*, two adjacent non-imprinted genes and two of three predicted enhancer elements downstream to *H19*. Methylation analysis by deep bisulfite next generation sequencing revealed hypermethylation of the maternal allele at the *IGF2* locus in both, mother and child, although *IGF2* is not affected by the deletion.

**Conclusions:**

We here report on a novel large familial deletion of the ICR1 region in a BWS family. Due to the deletion of the ICR1-DMR CTCF binding cannot take place and the residual enhancer elements have access to the *IGF2* promoters. The aberrant methylation (hypermethylation) of the maternal *IGF2* allele in both affected family members may reflect the active state of the normally silenced maternal *IGF2* copy and can be a consequence of the deletion. The deletion results in a variable clinical phenotype and expression.

**Electronic supplementary material:**

The online version of this article (doi:10.1186/s12881-015-0173-2) contains supplementary material, which is available to authorized users.

## Background

Beckwith-Wiedemann syndrome (OMIM 130650) is an overgrowth disorder characterized by exomphalos/omphalocele, macroglossia, gigantism and high risk for tumor development [1-3]. BWS has an estimated pan-ethnic incidence of 1/13,700. It is usually sporadically transmitted (85%), but familial occurrence has been reported in about 15% of cases [3]. Variations in clinical manifestations may be linked to molecular heterogeneity [4]. Different and complex molecular mechanisms have been implicated in the dysregulation of imprinted growth regulatory genes on chromosome 11p15 [5].

Evidence of three major or two major and three minor criteria usually supports the diagnosis of BWS. Major clinical findings includes among others: macrosomia, macroglossia, omphalocele or umbilical hernia, ear pits or anterior linear ear lobe creases, visceromegaly, adrenocortical cytomegaly, hemihyperplasia, renal abnormalities, and embryonal tumors. Minor signs are: polyhydramnios, neonatal hypoglycemia, characteristic facies, advanced bone age, naevus flammeus, cleft palate, rectus diastesis and cardiac anomalies [6].

Genomic imprinting is an epigenetic mechanism of gene modification, starting during gametogenesis and resulting in the differential expression of the paternal and maternal alleles during development. By this process, genes which are subject to genomic imprinting are expressed from one parental allele only. Deregulation of imprinted genes can lead to specific diseases. BWS syndrome is one of the most well-known human diseases implicated in genomic imprinting [7].

The 11p15.5 region contains two clusters of imprinted genes, which play a role in cell cycle and growth regulation [8,9]. Imprinted expression of these two gene clusters are regulated by two independent imprinting control regions, ICR1 and ICR2. ICR1 regulates the imprinted expression of genes in the most telomeric imprinted region including the insulin like growth factor 2 (*IGF2*) and *H19*. Molecular alterations affecting the ICR1 in patients with BWS lead to biallelic expression of *IGF2* and to loss of function of *H19*. ICR2 controls the imprinted expression of genes in the more centromeric region, such as cyclin- dependent kinase inhibitor 1C *(CDKN1C/p57*), potassium voltage-gated channel subfamily Q member 1 (*KCNQ1*) and KCNQ1- overlapping transcript 1 (*KCNQ1OT1* or *LIT1*) [8,9]. Molecular alterations in BWS affecting the ICR2 lead to silencing of *CDKN1C* and *KCNQ1* and biallelic expression of *KCNQ1OT1*. At least one of the many genetic/epigenetic alterations is recognized in ~ 80% of cases [9] and includes the following:

- Isolated hypomethylation of the KvDMR/ICR2 (~50-60%), which is implicated with the reduction of *CDKN1C/p57* expression
- Paternal uniparental isodisomy of 11p15.5 (~20%)
- Isolated gain of methylation of ICR1 and biallelic *IGF2* expression (2-7%)
- Chromosome rearrangements (dup(11)(p15) of paternal origin) (1-2%)
- Maternal *CDKN1C* mutations, representing 5-10% of sporadic cases and 4050% of familial cases
- Rare cases with maternal deletions affecting the ICR1 or ICR2 (less than 1%)
- Rare cases with small deletions or mutations affecting an OCT4 binding motif inside the ICR1-DMR between *H19* and *IGF2.*

Familial or *de novo* deletions in BWS affecting the ICR1 or ICR2 on chromosome 11p15 are rare findings. Only four maternal deletions affecting the ICR2 have been reported so far [10-13] However, in one case the deletion includes the *CDKN1C* gene, explaining the BWS phenotype because of the lack of the maternal *CDKN1C* transcript. All these ICR2 deletions are large in size spanning 198-900 kb genomic DNA. In contrast, most of the ICR1 deletions identified so far, are small microdeletions of 0.5 to 2.8 kb affecting CTCF binding inside the ICR1 [14-21]. For some of these deletions (sized 1.4 kb, 1.8 kb and 2.2 kb, respectively) it has been shown that the phenotypical outcome appears to depend on the spatial arrangement of the remaining CTCF-binding sites [21]. Only a few larger maternally inherited deletions in patients with BWS including *H19* in addition to the ICR1 have been described recently by Baskin et al. [19] and one paternally inherited deletion in a mosaic state, which interestingly lead to atypical Silver-Russel syndrome (SRS, an intrauterine growth retardation syndrome) [22,23].

Here, we present a novel familial case of BWS, in which the underlying molecular defect is a rare maternally transmitted deletion of ~100 kb in size. The deletion was found to be on the maternal chromosome 11 in the mother and her son. Both have BWS, although the clinical phenotype of the son was more severe than that of the mother. Hypermethylation of the DMR0 of *IGF2* in both affected family members, gives a hint for biallelic expression of *IGF2*, which is causative for the BWS phenotype.

## Case presentation

The male patient of Greek origin was the only child of the parents. He was born after 40 weeks of gestation through a caesarian section due to breech presentation [birth weight 3.250 (50^th^ centile), length 53.5 cm (>95^th^ centile), head circumference 34.6 cm (50^th^ centile)]. The Apgar score was 8 at 1′ and 9 at 5′. The parents were non-consanguineous (mother was 35 and father was 36 years old) and the conception was not assisted. The pregnancy and the prenatal ultrasound were reported as normal. The mother of the child had macrosomia (height = 95^th^ centile), large hands and feet with lymphedema of the lower limbs. The mother’s phenotype from her childhood was suggestive of the clinical diagnosis of BWS (gigantism, macroglossia and small omphalocele). The patient’s father, the maternal grandmother and grandfather were reported as phenotypically normal.

**Figure 1:**
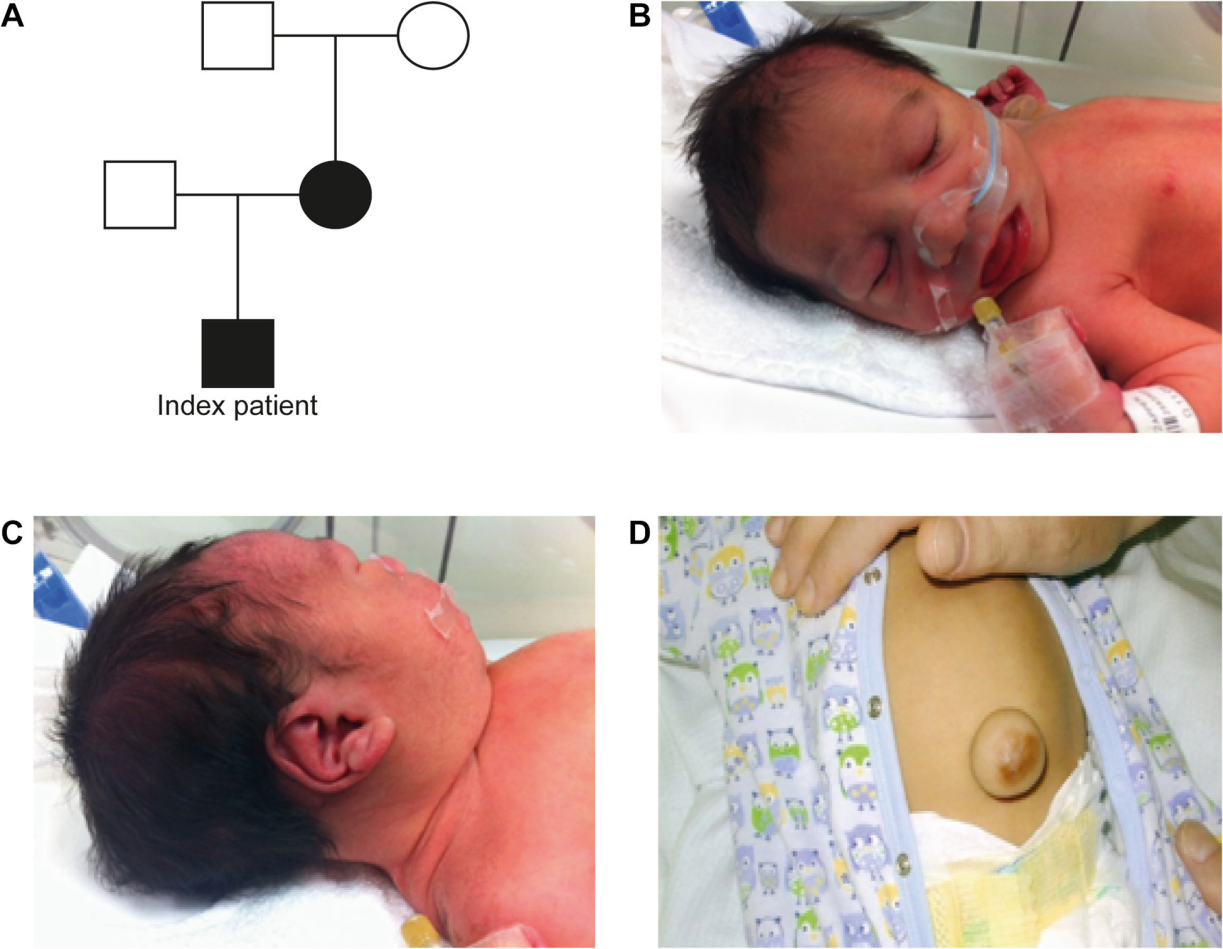
Patient **A**. Pedigree of the BWS family. Deletion carriers are indicated by filled circles or boxes. **B**. Frontal view of the newborn patients face **C**. Linear crease of the ear lobe **D**. Presence of omphalocele at 1 month.

At birth, the newborn was hypotonic with facial BWS dysmorphisms, macrosomia but with no obvious omphalocele. There was a small palpable mass at the sacrococcygeal region (Figure 1A, B, C). In the first 24 hours, the patient presented with hypoglycemia attributed to hyperinsulinism and was treated with diazoxide. The hypoglycemia was persistent and intractable. From the biochemical screening, in addition to hypoglycemia, the patient had AFP (60.500 ng/ml-normal range maximum 40.000), liver function tests (transaminases) and direct bilirubin levels which were remarkably elevated, but with normal ammonia levels. The cardiac ultrasound revealed an atrial septal defect with mild hypertrophy of the septum and the right ventricle, along with mild bilateral stenosis of the pulmonary arteries. TORCH and metabolic investigations were negative. Due to the intractable hypoglycemia, a therapeutic trial with somatostatin was also attempted, but without relevant clinical benefit. At the age of two months, the proband was still mildly hypotonic, had macrosomia, a notable umbilical hernia, macroglossia, and hypertelorism with a flat nasal bridge, all of which are clinical features of BWS. Heart ultrasound of the patient remained the same as during the first 24 hours. Abdominal ultrasound and Magnetic Resonance Imaging (MRI) confirmed the hepatomegaly but without dilatation of the intra and extrahepatic biliary vessels, the splenomegaly, a normal pancreas, adrenals and enlarged but otherwise morphologically normal kidneys. An X-ray of the left hand, also taken at two months, revealed advanced bone age (one year more than his chronological age). Due to the presence of a small sacrococcygeal mass, MRI of the lower spine revealed spina bifida at the level of I_1_-I_2_ vertebra. Because of the macroglossia, the patient developed severe upper airway congestion and was placed on mechanical ventilation (CPAP). After eight days his blood sugar stabilized. After 5 months of hospitalization, his AFP levels dropped and he could eat and breathe by himself. After clinical reevaluation at the ages of 8, 12 and 15 months his macrosomia remained out of the 97^th^centile (above 2 SDs). At 15 months his developmental milestones were slightly delayed (he walks with support) but he did not present a neurological deficit.

### Cytogenetic analysis

Conventional karyotyping by G-banding technique was carried out on stimulated peripheral blood cultures on metaphase cells according to standard protocol. Fifty metaphases with 550 band resolution (≥5 Mb) were analyzed.

### DNA extraction

Genomic DNA from peripheral blood was extracted according to standard protocols (QIAGEN BioRobot M48, MagAttract DNA Blood Kit, Hilden, Germany).

### Methylation-specific multiplex ligation-dependent probe amplification (MS-MLPA)

Gene dosage and methylation of the *H19*/ICR1-DMR/*IGF2* and ICR2 on chromosome 11p15 were analyzed by MS-MLPA using the SALSA MLPA KIT ME030-C3 BWS/RSS (MRC Holland, Amsterdam, Netherlands). Hybridization, ligation and PCR reactions were carried out according to the manufacturer’s instructions. Amplification products were analyzed by capillary electrophoresis using the ABI3100 or 3500 capillary sequencer, respectively. Data analysis was carried out using the Gene Marker Software (Softgenetics, State College, PA, USA).

### Bisulfite treatment of DNA

Bisulfite treatment of genomic DNA was carried out using the EZ DNA MethylationGold Kit (Zymo Research Europe, Freiburg, Germany) according to the manufacturer’s protocol.

### Deep bisulfite sequencing

Generation of bisulfite amplicon libraries, sample preparation and sequencing on the Roche 454 GS junior system was carried out as previously described [21,24]. For the *IGF2* DMR0 following primers were used (product size 349 bp; Tm = 62°C): IGF2-DMR0-Ftag 5′-**CTTGCTTCCTGGCACGAG-**AGATTTTTTTGTTGGATAGGTTGTT-3′, IGF2-DMR0-RM13 5′- **CAGGAAACAGCTATGAC**TCTATTACACCCTAAACCCAAACTC -3′ (in **bold** universal tags). For allele separation we used the SNP rs3741210 (A/G). For data analysis, we used the Python-based amplikyzer software developed in-house [25].

### Molecular karyotyping

Array analysis was carried out using the genome-wide high-resolution SNP array CytoScan HD (Affymetrix) according to the manufacturer′s protocols (Affymetrix, SantaClara, California, USA). Evaluation of the data was performed using the Affymetrix Chromosome Analysis Suite version 2.0.

### In silico enhancer characterization

Candidate enhancer sequences of the *IGF2/H19* genes have been identified through bioinformatics analysis for the presence of the following characteristics: 1) conserved non coding regions of at least 100 bp (>70% identical between human and rodents); 2) enriched histone H3K27 acetylation and H3K4 monomethylation; 3) DNase hypersensitive sites and transcription factor binding [26-28].

### Results

Cytogenetic analysis had revealed a normal karyotype in both, the mother and the patient. Because of the clinical diagnosis of BWS in the propositus and his mother, a combined gene dosage and methylation analysis by MS-MLPA for the chromosome 11p15 imprinted region was performed in both. In addition, the father of the propositus and the maternal grandparents were investigated by MS-MLPA. By this method, a deletion affecting all nine of the ICR1-DMR and *H19* specific MLPA probes was detected in the patient and his mother. All four methylation-specific MLPA probes for the *H19*/ICR1-DMR showed complete methylation in both affected individuals An additional excel file presents detailed MLPA findings (see Additional file 1: Table S1). In contrast, all other studied family members showed a normal MLPA pattern. The aberrant methylation pattern in both affected individuals indicated that both have the deletion on their unmethylated, i.e. maternal chromosome explaining the BWS phenotype. None of the MLPA probes for *IGF2* or the ICR2 showed a reduction in the gene dosage, indicating that the deletion is restricted to the ICR1-DMR, *H19* and probably to the downstream region of *H19*. Since the maternal grandmother has no deletion (data not shown), the deletion in the mother seems to be a *de novo* event; although a germline mosaicism for a deletion in the grandmother cannot be excluded.

**Figure 2:**
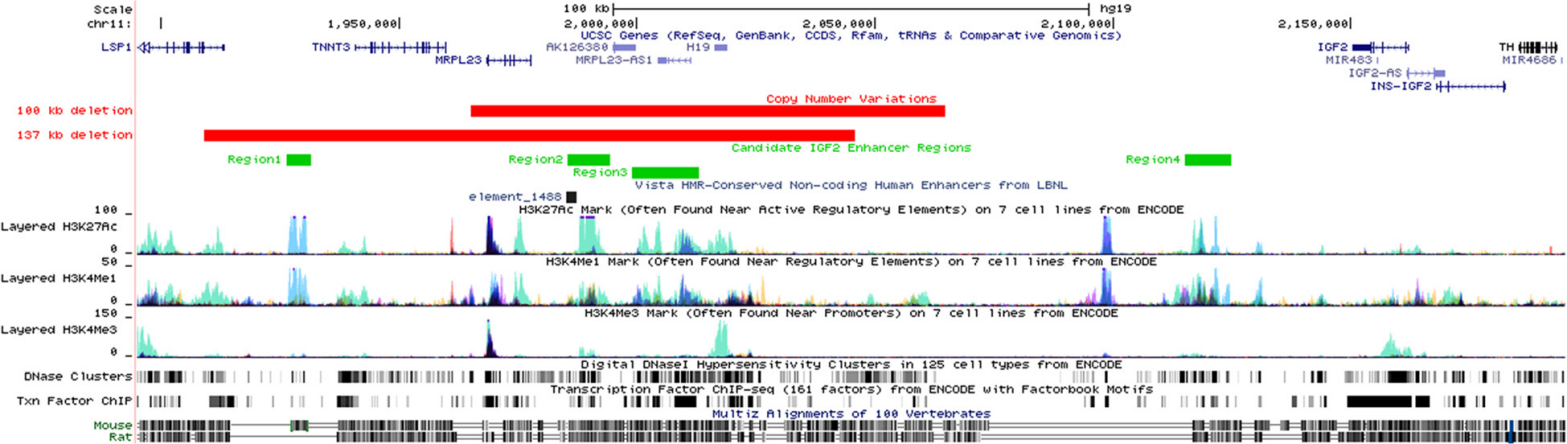
Overview of *H19/IGF2* genomic region on 11p15.5 including deletions and putative enhancer elements. Identification of candidate *IGF2* enhancer regions through the integration of data of chromatin features and sequence conservation at 11p15.5. The image shows an UCSC Genome Browser screen shot including the most relevant information used to locate the putative enhancers. Two custom tracks have been added: the copy number variations track showing the extension of the 100 kb deletion described in the paper and the 137 kb deletion described by Baskin et al., 2014 (red bars); the putative enhancers of *IGF2* showing four candidate regions represented by green boxes. Note that these regions correspond to evolutionary conserved intronic regions that are enriched in H3K27Ac, H3K4me1, DNase hypersensitive sites and transcription factors binding as reported by ENCODE (http://genome.ucsc.edu Chr11: 1,895,000-2,195,000; GRCh37/hg19 Feb 2009 assembly).

To further narrow down the breakpoints of the identified deletion in 11p, we performed genome wide array analysis in the patient. By this approach we determined the size of the deletion to at least 99.78 kb and at most 100.02 kb. The deletion was described as arr[hg19] 11p15.5 (1,965, 037×2, 1,965, 0472, 064, 827×1, 2,065, 052×2). The deletion encompasses the *H19*, *miR-675*, the ICR1DMR and the adjacent non-imprinted genes *MRPL23*, *MRPL23-AS1*, but not *IGF2* or the ICR2 region (Figure 2). The centromeric breakpoint is estimated to map ~52 kb downstream of *H19*. The telomeric breakpoint maps approximately 46 kb upstream of *H19* and therefore 90 kb downstream of *IGF2*.

Mouse transgenic studies have identified multiple shared enhancers for *Igf2* and *H19* between 10 and 120 kb 3′ of *H19* [29]. Enhancer sequences are characterized by conserved non coding regions that are enriched in specific histone modifications (i.e. H3K27acetylation, H3K4monomethylation), DNase hypersensitive sites and transcription factor binding [28,30]. We identified four sequences meeting these features at the *IGF2/H19* locus (Figure 2). Three of them are located 85, 26 and 10 kb at the 3′ of *H19*, respectively, reflecting the position of murine enhancers that have been identified by transgenic mouse studies [29]. A fourth region with enhancer features is located in the intergenic region between *IGF2* and *H19*, at ~100 kb far from *H19*. Two of these sequences are located within the region deleted in our family.

**Figure 3:**
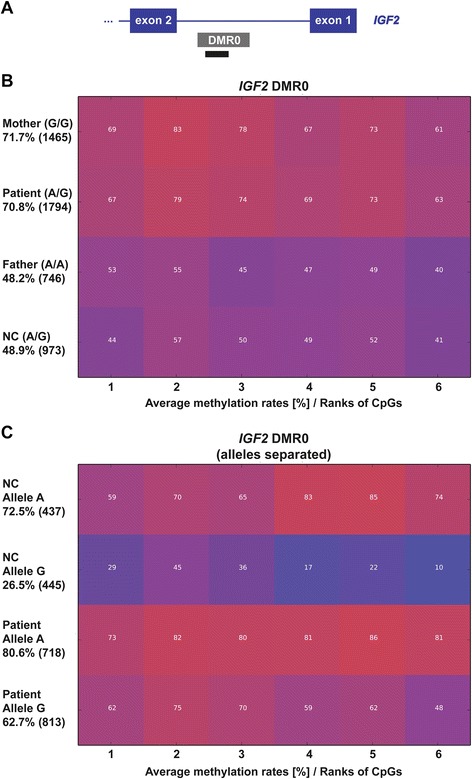
Comparative methylation plots and heatmaps for the *IGF2* DMR0. **(A)** Schematic view of the *IGF2* DMR0 and the location of the amplicon used for deep bisulfite sequencing (black bar; not drawn to scale). The exact position for the amplicon is chr11:2169292-2169605 (hg19, UCSC Browser). **(B)** The average methylation of the *IGF2* DMR0 in the patient, his parents and a normal control. Compared to the father and the normal control (NC) the mother and the patient show a hypermethylation for the *IGF2* DMR0. **(C)** The average methylation of the separated alleles (SNP rs3741210) in a normal control and the patient. One allele of the normal control is hypermethylated (indicating that it represents the paternal allele), whereas the other allele is hypomethylated (indicating that it representing the maternal allele). In the patient the paternal allele **(A)** is hypermethylated and the maternal allele (G), which should be hypomethylated, is hypermethylated as well. Each square represents a CpG dinucleotide with its average methylation level, each line a specific sample. The average methylation over the analyzed region is given in percentage on the left under the sample ID together with the number of analyzed reads in brackets. The methylation was analyzed over 6 CpGs.

Since no probe for the *IGF2* locus to test differential methylation was included in the MS-MLPA kit, we performed next generation bisulfite sequencing of the DMR0 of *IGF2* in blood DNA from the mother, the father and the son and an unrelated normal control. Normally this DMR0 is hypermethylated (~70%) on the active paternal allele of *IGF2* and hypomethylated (~30%) on the silent maternal allele [31] (and own unpublished data). We found a hypermethylation of the DMR0 in the mother and her son compared to the healthy father and the normal control. For the six CpG dinucleotides studied, the mother showed an average methylation of 71.7% and her son an average methylation of 70.8%, while the average methylation in the father and the normal control (NC15) was 48.2% and 48.9%, respectively (see Figure 3B). Since the patient and one of the normal controls were found to be heterozygous for a single nucleotide polymorphism (SNP, rs3741210, A > G) we were able to separate the parental alleles. Since the mother of the patient is homozygous for the G allele and the father is homozygous for the A allele, the parental origin of the two alleles in the patient could be determined. By this we could show that the normal control has one allele which is hypermethylated (72.5%) whereas the other allele is hypomethylated (26.5%, see Figure 3C). In contrast, in the patient both alleles (A and G) show a hypermethylation of 80.6% for the paternal allele (A) and 62.7% for the maternal allele (G), indicating that the maternal deletion allele acquired methylation at the DMR0 although the deletion does not overlap *IGF2*.

Our findings are in agreement with the results published by Murrell et al. [31].

## Conclusions

BWS results from deregulation of imprinted genes on chromosome 11p15 and is known to have clinical variability and molecular heterogeneity. There are phenotypic differences between the various molecular subgroups of BWS [9]. Familial or *de novo* deletions affecting one of the two ICRs in 11p15 are a rare cause of BWS. Only 14 deletions for ICR1 have been described so far [14-21,23]. Ten of these deletions are very small and affect only the ICR1 itself. These deletions affect multiple target sites (CTSs) for the zinc-finger protein CTCF, whose binding on the maternal allele blocks the access of the *IGF2* promoter to its downstream enhancers and enables *H19* to use these enhancers for transcription. The ICR1-DMR deletions affect the ICR1 function and CTCF binding to different extents resulting in variable expression of the clinical phenotype depending on the spatial arrangement of the remaining CTSs [21]. Of the four larger deletions resulting in BWS after maternal transmission reported by Baskin et al. [19] three (18-25 kb) include the *H19* gene in addition to the ICR1 and one deletion of 137 kb include the non-imprinted genes *MRPL23*, its antisense *MRPL23-*AS1, *TNNT3* and part of the *LSP1* gene downstream of *H19*. However, none of these deletions include *IGF2*. The deletion in our family is ~100 kb in size and includes *H19*, the *miR-675*, which is located inside *H19*, the non-imprinted gene *MRPL23,* its antisense transcript *MRPL23-AS1* and two of three putative enhancer elements downstream of *H19*.

*MRPL23* has previously been shown to be functionally insulated from the *IGF2/ICR1/H19* domain in terms of both imprinting and enhancer action [32]. Thus, a heterozygous deletion of *MRPL23* should not give rise to any of the BWS phenotypic features present in the patient and the mother.

From mouse models but also from the deletion cases with small deletions inside the ICR1-DMR it is known that maternal transmission of a ICR1-DMR deletion itself leads to reactivation of the maternal *IGF2* copy and therefore leads to biallelic expression of *IGF2* resulting in BWS [14,20,29] Reactivation of *IGF2* is associated with hypermethylation of the maternal allele [31]. We could demonstrate that the deletion leads to a gain of methylation of the maternal allele at the DMR0 of *IGF2* in both, the patient and his mother. The DMR0 is located 5′ to the main promoters of *IGF2*. In 2008 Murrell and coworkers demonstrated that the *IGF2*-DMR0 is subjected to different methylation changes in cancer and congenital growth disorders [31]. In normal peripheral blood and kidney the DMR0 is predominantly methylated on the paternal allele and unmethylated on the maternal allele. In addition, BWS patients with and without microdeletions have *IGF2*-DMR0 as well as ICR1 hypermethylated while patients with Silver-Russell syndrome (SRS), a growth retardation syndrome, have hypomethylation at both of these sequences [31,33]. From these data it has been concluded that in early embryonic development the *IGF2-*DMR0 is methylated or unmethylated according to the germline methylation imprint of the ICR1-DMR. Gain of methylation of the DMR0 leads to activation of the maternal *IGF2* allele in ICR1 hypermethylated BWS cases and loss of methylation of the DMR0 leads to silencing of the paternal *IGF2* allele in ICR1 hypomethylated SRS cases [31].

In the family presented here, the deletion patient and his mother showed hypermethylation on the maternal deletion allele at the *IGF2* DMR0, indicating that the maternal allele is transcriptionally active. In this case, the hypermethylation does not reflect the methylation status of the ICR1-DMR since this DMR is deleted. However, it may reflect the active state of the *IGF2* promoters and possibly arises as a consequence of the interaction of the *IGF2* promoters to its downstream enhancers. The exact location of these downstream enhancers is well known in mice but largely unknown in human [29]. By *in silico* analysis we could identify four candidate regions for putative enhancers. Two of these sequences are affected by the deletion in our family. From the methylation status of *IGF2* it seems that the residual two enhancer elements are sufficient to drive *IGF2* expression on the maternal allele. Moreover, evidence that only the most telomeric and/or the most centromeric putative enhancer elements are needed to activate *IGF2* comes from a patient with the only similar deletion of 137 kb in size reported by Baskin and colleagues [19]. Compared to our deletion, this deletion includes one additional putative enhancer element downstream of *H19* (see Figure 2). Although methylation or expression studies for *IGF2* have not been performed in this patient, there must be an activation of the maternal *IGF2* allele since the patient has phenotypic features of BWS. However, Gronskov et al. [23] recently reported an atypical SRS patient with a deletion in mosaic on the paternal chromosome 11, which is similar to the deletion in our patient. The telomeric breakpoint of this deletion is nearly the same whereas the centromeric breakpoint is closer to *H19* and therefore spans also the same enhancer elements as the deletion in our patient. It is interesting that a deletion of *H19* and its enhancers, in opposite inheritance, can give rise to either atypical SRS or BWS. Furthermore, Gronskov et al. could show that in the SRS patient methylation of the *IGF2* DMR0 is slightly changed to hypomethylation. The fact that aberrant methylation in this patient is not very strong may be due to the mosaic state of the deletion in this patient.

It is known that *IGF2* has a key role in fetal and placental development by activating intracellular signaling cascades that promote cell growth and survival.

Interestingly, in a recent study it has been reported that the methylation level of *IGF2*DMR0 but not DMR2 is significantly increased in brain tissues of neural tube defect fetuses and that a hypermethylation of the *IGF2*-DMR0 is positively associated with an increased risk of neural tube defects [34]. This might explain the presence of spina bifida in the lower back of our deletion patient, a feature which is not listed in the clinical features of BWS [34]. However, alterations affecting the ICR1-DMR and therefore in turn affecting *IGF2* methylation and expression are only found in a minority of patients with BWS (~10%), making a judgment of increased spina bifida occurrence difficult. *IGF2* upregulation is also known to be associated with an increased risk for embryonal tumors including Wilms tumor. Thus, the occurrence of embryonal tumors in early life is a major concern in patients with BWS and ICR1/*IGF2* alterations. Our patient has not yet developed Wilms tumor but since he is at high risk for tumor development ultrasound surveillance is being performed in 3 month intervals until the age of 6 years.

Although the patient and his mother have the same deletion, the clinical phenotype of the patient is more severe than that of his mother. For example, he was severely affected by hyperinsulinaemic hypoglycemia which was not noted in his mother. A multidisciplinary approach was necessary to prevent the threat on the patient’s life. It is not known how severely the patient with the 137 kb deletion [19] was affected, since clinical data for this patient have not been reported.

An increased clinical severity can be a random result of variable phenotypic expression, which is not uncommon in BWS. Evidence for anticipation in BWS in members of a family with a maternally inherited *OCT4* binding site mutation has recently reported by Berland et al. [24]. Two sisters and their cousin had classical BWS with Wilms tumor whereas their mothers and a third sister of the mothers have only tall stature and only one of these sisters showed also mild BWS features as a child. In this family the increased clinical severity from one generation to the next correlates with increased methylation of the maternal ICR1-DMR. However, all these individuals represent methylation mosaics with a gain of methylation on their maternal chromosome. Since the maternal ICR1-DMR in our patient and his mother is deleted an increased clinical severity could not be linked to methylation differences of this DMR on the maternal chromosome. In contrast, the hypermethylation of the *IGF2*-DMR0 is similar in the patient and his mother and could not explain the variable clinical expression. However, this does not exclude differences in methylation in tissues other than blood.

We report here a novel familial deletion encompassing most of the imprinted ICR1 region on chromosome 11p15. Both deletion carriers, the mother and the son, have BWS with variable clinical expression, but similar hypermethylation at *IGF2* DMR0 in peripheral blood. *In silico* analysis revealed that two of three putative enhancer elements downstream of *H19* are affected by the deletion, suggesting that the remaining two enhancers are sufficient to drive *IGF2* expression on the maternal chromosome.

## Consent

Written informed consent was obtained from the parents for publication of this case report and any accompanying images. A copy of the written consent is available for review by the Editor of this journal.

### Ethics statement

The approval was obtained from the Ethics Committee of “Aghia Sophia” Children’s Hospital, Athens, Greece.

## Additional file

Additional file 1: Table S1. Results of the MS-MLPA analysis. Gene dosage analysis revealed that the patient and the mother have a 50% reduced dosage for all H19/ICR1 probes of chromosome 11p11.5 (dark grey shaded), whereas all other probes for chromosome 11p11.5 (light grey shaded) and all reference probes from other chromosomes showed a normal dosage. Methylation analysis showed complete methylation of approximately 100% in both the patient and his mother for the four methylation sensitive H19 probes, which lie inside the deletion, indicating that they have the deletion on their unmethylated maternal allele. The methylation specific probes for the ICR2 region, which is not affected by the deletion, showed a normal methylation of ~50%. P, patient; M, mother.
